# 宏基因组二代测序技术在血液病患者感染病原诊断中的应用中国专家共识（2023年版）

**DOI:** 10.3760/cma.j.issn.0253-2727.2023.08.001

**Published:** 2023-08

**Authors:** 中华医学会血液学分会抗感染学组

血液病患者因原发病和（或）治疗相关原因等，免疫功能低下，感染是其常见的并发症[1]–[2]。血液病患者感染相关临床症状和体征常不典型，感染病原谱广，传统微生物学检测阳性率低、耗时长。因此，病原诊断是优化抗感染治疗、改善患者预后的关键环节。病原宏基因组二代测序（mNGS）是一项覆盖病原谱广且高通量的检测技术，已在临床感染领域得到了广泛的应用。在血液病患者感染病原诊断方面，mNGS检测具有阳性率高、受到抗菌药物干扰小、覆盖病原广的优势。然而，目前尚无mNGS在血液病感染病原诊断中应用的指南或专家共识。为优化血液病患者病原mNGS检测适应证及规范报告解读，在参考血液病领域感染诊治及病原mNGS相关指南[1],[3]–[4]等的基础上，中华医学会血液学分会抗感染学组特邀请血液学、病原mNGS检测等领域专家共同制定此专家共识。

一、血液病患者感染临床送检mNGS适应证

共识1：感染是血液病患者常见的并发症，当疑似感染发生时，应首先选择传统微生物学检测，仅在特殊情境下谨慎选择病原mNGS检测（图1）。

**图1:**
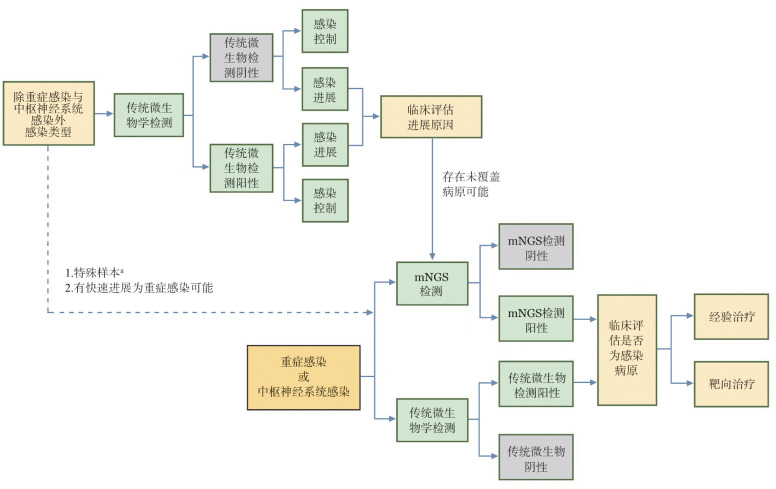
血液病患者病原宏基因组二代测序（mNGS）送检流程图 **注** ^a^特殊样本包括组织、骨髓、穿刺液、支气管肺泡灌洗液等无菌来源或不易重复获取的样本

（一）中性粒细胞缺乏（粒缺）伴发热

共识2：低危且无明显感染灶的患者，如经验抗菌药物治疗≥7 d未明显好转可考虑在送检血培养的同时送检外周血标本mNGS；高危患者初始经验性治疗72～96 h无效时，推荐送检传统微生物学检测的同时送检血液mNGS。

多项研究显示：在粒缺伴发热患者感染中mNGS检测灵敏度高于传统微生物学检测，尤其对于已经接受经验性治疗的患者，传统微生物学检测可因抗菌药物使用灵敏度明显降低，而mNGS受影响程度小于传统微生物学检测；与此同时，mNGS对于识别病毒、真菌以及多种微生物混合感染具有优势[5]–[7]。

（二）血流感染（BSI）

共识3：疑似BSI患者，在留取血培养标本的同时留取血浆样本，于−80 °C暂存；如72 h内血培养阴性且抗感染治疗无改善的患者，推荐将留存样本进行mNGS。疑似脓毒症的重症患者，建议送检血培养同时送检血mNGS。

多项研究显示：联合mNGS与血培养可以显著提高病原检出率，同时增加混合感染病原的检出；与此同时，研究表明在已明确病原的BSI中，病原的持续检出可与BSI严重程度及控制不佳有关[8]–[10]。在导管相关BSI中，凝固酶阴性葡萄球菌、棒状杆菌是常见的感染病原，因其也是皮肤定植菌，mNGS检出时难以区分定植与感染。因此，不常规推荐mNGS作为疑似导管相关BSI的首选送检方式。

（三）下呼吸道感染

共识4：下呼吸道感染患者首选支气管肺泡灌洗液（BALF）送检传统微生物学检测，难以进行支气管肺泡灌洗的患者，可以选择深部痰进行检测，并同时留取标本冻存。如抗感染治疗≥72 h感染症状无好转或影像学表现持续加重的患者，建议送检留存标本行mNGS检测。如为重症下呼吸道感染或有快速进展为重症的危险因素患者，建议同时送检传统微生物学检测及mNGS。

在病原谱上，细菌以革兰阴性杆菌为主，尤其是非发酵革兰阴性杆菌；真菌主要包括曲霉属、毛霉目和耶氏肺孢子菌等；病毒主要包括呼吸道病毒、巨细胞病毒（CMV）、腺病毒等；同时，混合感染并非少见[5],[11]–[12]。鉴于血液病患者肺部感染病原谱广，部分病原难以培养或传统检测不能覆盖，且混合感染常见，推荐对于经验性抗感染治疗无效及重症下呼吸道感染患者在完善传统微生物学检测的同时，采用BALF mNGS进行病原诊断。

血液mNGS对于下呼吸道感染病原诊断效果局限[13]–[15]。有研究显示毛霉目为病原的肺感染，外周血mNGS检测灵敏度较高，而曲霉属感染灵敏度较低。对于细菌或病毒性下呼吸道感染，外周血与BALF相比，病原检出灵敏度低且病原分布明显不同，单次血液mNGS检测的灵敏度并不优于呼吸道样本传统微生物学检测。

（四）中枢神经系统感染（CNSI）

共识5：疑似CNSI，推荐脑脊液在送检传统微生物学检测的同时送检mNGS，在流程上建议选择DNA检测，仅在考虑RNA病毒感染时完善RNA检测流程。

病毒是异基因造血干细胞移植（allo-HSCT）受者CNSI的主要病原，包括：人类疱疹病毒6型（HHV-6）、单纯疱疹病毒（HSV）、Epstein-Barr病毒（EBV）、CMV、水痘带状疱疹病毒（VZV）等[16]–[17]。部分CNSI病原如脑膜炎奈瑟菌、肺炎链球菌、流感嗜血杆菌、类鼻疽伯克霍尔德菌、产单核李斯特菌、HSV及流感病毒等，其感染路径可不经过血行播散，对于这类病原引起CNSI时，血液中可无病原检出。疑似CNSI，应首先完善传统微生物学检测及多重病毒分子生物学检测，如所在单位开展项目局限，建议同时送检脑脊液进行传统微生物学及mNGS检测。国内刘启发教授团队对allo-HSCT受者发生CNS并发症前瞻性研究显示，脑脊液mNGS检测有助于提高病原检出率且灵敏度高于传统微生物学检测[18]。

脑脊液作为无菌样本，受定植干扰较小，mNGS检测出微生物应充分考虑其是病原可能，不推荐将传统微生物学检测阴性作为排除感染的标准。同时，mNGS作为一种定性检测，序列数目并非等同于载量。对于EBV、CMV等潜伏病毒，检出不等同于感染，需结合临床进行解读。此外，CNSI时外周血与脑脊液病原分离现象（脑脊液存在感染病原，而外周血不能检出）并不少见。因此，对于疑似CNSI患者，不推荐外周血作为替代样本进行mNGS检测。

（五）肠道或腹腔感染

共识6：疑似肠道感染患者推荐首选传统微生物学检测，粪便样本病原mNGS检测可作为补充。疑似腹腔感染，首选感染部位样本送检，如不能获得感染部位样本可选择血液样本送检，但检出性能有限，不作为常规推荐。

目前尚无针对血液病患者肠道或腹腔感染病原mNGS检测研究报道。由于肠道菌群定植率高，在报告解读分析病原上存在难度。对于常见引起肠道感染的致病微生物如沙门菌、艰难梭菌、霍乱弧菌有临床意义，而对于肠道常见定植菌检出临床意义有限。对于病毒的检出，如为胃肠炎病毒（轮状病毒、肠道腺病毒、星状病毒）检出往往是致病病原。对于腹腔感染，如患者已进展为脓毒症休克，推荐首选血液样本mNGS，而相对症状较轻的患者可以选择腹水或血液样本送检。

（六）皮肤软组织感染

共识7：推荐对累及深部或播散性的皮肤软组织感染患者，在完善感染部位标本传统微生物学检测的同时送检mNGS，如不能获得感染灶局部样本，可选择外周血。

皮肤软组织是血液病患者感染常见的累及部位，这类感染容易继发播散，同时也可为播散性感染的表现之一。引起感染的细菌包括金黄色葡萄球菌、铜绿假单胞菌、嗜麦芽窄食单胞菌、气单胞菌属、诺卡菌属、非结核分枝杆菌等；病毒以人类疱疹病毒最为常见，潜伏性感染的重新激活是病毒性皮肤软组织感染的最常见原因，主要包括HSV-1、VZV等；真菌主要包括镰刀菌、念珠菌、毛孢子菌等。

mNGS对于皮肤软组织感染的优势在于阳性率高、覆盖谱广及对混合感染的识别能力强[19]。在送检时，应首选局部样本，其病原检出率高于血液样本。对于播散性感染，在不能获得局部样本时可选择外周血作为替代或根据临床具体情况选择感染部位以及血液样本平行送检。

（七）泌尿系统感染

共识8：对于复杂性泌尿系统感染、移植后泌尿系统感染在完善传统微生物学检测后不能获得病原学证据或治疗不佳的患者可尝试尿液mNGS。

常见感染病原包括肠杆菌科细菌、肠球菌、酵母菌、病毒等[20]。目前尚无mNGS应用于血液系统疾病患者泌尿系统感染的研究，但在泌尿系统感染中，mNGS对于培养阴性、混合病原感染、慢性尿路感染具有优势[21]–[22]。对于传统微生物检测阴性的泌尿系统感染的患者，可尝试进行尿液mNGS，如感染灶在肾脏，首选穿刺采集组织送检。

二、样本采集与质量控制

共识9：对于病原mNGS检测样本，应首选从感染部位直接采集，若无法采集可考虑外周血标本送检。

推荐首选病变部位穿刺或手术采样，如不能获得该类样本，在选择邻近部位样本时应尽量减少正常菌群和定植菌的干扰。在样本采集后应尽快转运至实验室进行检测，以减少核酸降解[23]。不同样本采集及注意事项见表1。多种感染类型血液中病原载量均低于感染部位样本，所以仅推荐血液作为替代样本送检。对于无明显感染灶、感染部位样本取材困难、经临床及实验室评估存在病原和（或）核酸入血的情况可进行血液mNGS。mNGS检测结果依赖于标本质量，标本采集与运输中应严格执行无菌操作与尽快转运原则。液体样本一般送检第二管以减少穿刺部位皮肤定植菌干扰。

**表1:** 宏基因组二代测序（mNGS）感染标本送检类型及注意事项

标本类型	标本量	采集容器	采集注意事项
外周血	5 ml	游离核酸保存管	1. 采血时避免与脂肪乳同时、输液侧同侧采集；采血后立即温和颠倒混匀8~10次，减少溶血 2. 血液标本采集前，应严格做好穿刺点及周边皮肤的清洁和消毒，尽可能减少皮肤定植菌对mNGS检测的影响
石蜡切片	10~15片	不适用	无
脑脊液	2 ml	无菌螺纹管	为减少定植菌污染，推荐收集第二管脑脊液进行送检
房水	≥0.2 ml	无菌螺纹管	无
关节液、穿刺液	2 ml	无菌螺纹管	关节液或穿刺液留取时推荐弃去最开始的2～3滴后留取
脓液	2 ml	无菌螺纹管	无
组织	≥3×3×3 mm^3^	无菌螺纹管	深部组织推荐采集病变部位；皮肤及软组织优先选择感染基底部位的组织，其次为基底部位的穿刺标本（液）；新鲜组织采集后不可添加福尔马林；若穿刺组织过小有干燥可能，可添加少量保护液或无菌盐水浸润组织
胸水、腹水	5~10 ml	无菌螺纹管	为减少定植菌污染，推荐收集第二管进行送检，引流袋内液体不推荐送检
尿液	5~10 ml	无菌螺纹管	清洁外阴后，采集清洁中段尿；推荐晨尿送检
肺泡灌洗液	5~10 ml	无菌螺纹管	为减少定植菌污染，推荐收集第二管进行送检
粪便	3~5 ml	无菌螺纹管	无
痰液	3~5 ml	无菌螺纹管	漱口2～3次，再用力咳出深部痰液
拭子	2~4支	无菌拭子	拭子采集应在清洁创面后尽可能取感染基底部位

**注** 所有标本采集后原则上均应立即送检；若需暂存，储存与运输条件为血、石蜡切片标本可室温暂存运输，其他标本−20 °C保存不超过1周，−80 °C长期储存，干冰运输；标本转运时避免标本反复冻融，避免剧烈震动

三、报告解读

共识10：致病性明确且罕见定植的微生物，低序列检出也应考虑其为致病菌可能。

对于明确为致病菌，尤其是可导致免疫功能健全人群感染的微生物例如结核分枝杆菌、隐球菌、嗜肺军团菌、鹦鹉热衣原体、寄生虫（血液病患者以弓形虫最为常见）等，这些病原不属于定植菌且致病性明确，在标本中经mNGS检出应充分考虑其是感染病原的可能性，同时建议进行其他传统微生物学检测加以验证，如PCR、培养、抗原抗体检测等。不同微生物在不同样本中的临床意义见图2。

**图2:**
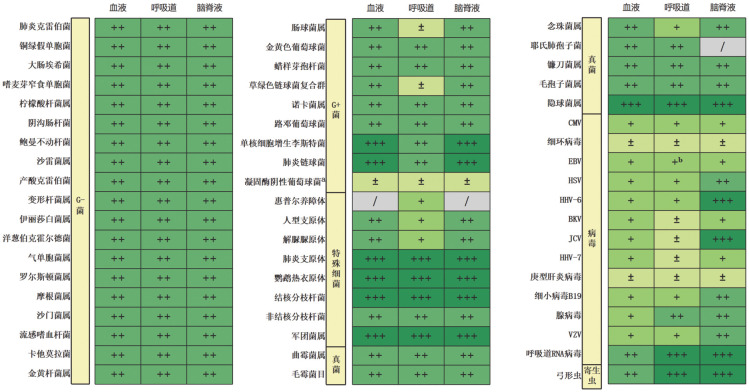
不同微生物在血液病患者常见临床样本中检出的临床价值判断 **注** /：该类感染未见报道或该病原罕见检出；±：定植或无明确致病意义；+：条件致病菌，可为感染病原同时也可存在无症状检出，建议结合临床与传统检测共同参考；++：条件致病菌，多数检出为感染病原；+++：检出应高度考虑是致病病原，传统微生物检测阴性不能除外感染。^a^多数凝固酶阴性葡萄球菌可为定植菌或背景菌，少数除外（如路邓葡萄球菌）；^b^ EBV引起的肺炎罕见，造血干细胞移植后EBV检出可与罕见的肺部淋巴组织增生性疾病有关

共识11：无菌采集获得的样本检出病原，应结合临床考虑致病微生物的可能，但需注意排除定植。

对于外周血、组织、脑脊液、胸腹水及其他穿刺液等正常无菌样本，mNGS检出的细菌与真菌应结合当地感染流行病学、患者感染部位及定植情况考虑其是否为致病病原。样本中单一高序列细菌或真菌检出往往代表其是致病微生物可能性较高，而对于多种微生物检出，其解读应结合检出病原的常见定植部位及可能的微生物或其核酸的入血途径进行判断。例如，肠道或腹腔感染时，肠道黏膜屏障的破坏可引起多种肠道微生物（或其核酸）入血，血液mNGS可出现多种微生物检出的可能，一方面不能除外发生混合病原BSI，另一方面也可能仅为核酸入血。

共识12：外周血样本mNGS报告中DNA类病毒检出常见，并非均为感染病原。

血液病患者mNGS报告中病毒检出常见，且往往可有多种病毒检出，并非均是感染病原，同时，病毒检出不等同于病毒复制，需结合核酸定量PCR检测评估其载量。如mNGS阳性但定量PCR阴性，需谨慎判断为病原的可能。

共识13：非无菌样本的结果应结合临床、标本类型、传统微生物学检测结果共同判断。

对于可存在定植微生物检出的非无菌样本如痰液、感染部位拭子、粪便等，mNGS检出的微生物在判断是感染、定植或污染时需要结合患者的临床表现、影像学特征和其他检验结果，一些病原的检出存在是定植菌的可能。例如耶氏肺孢子菌可在呼吸道中定植，因此呼吸道样本mNGS检出耶氏肺孢子菌不等同于感染，需要结合临床综合判断[24]–[25]。

共识14：耐药基因的检出，应在充分考虑基因型与耐药表型对应性的基础上进行判断。

mNGS在一定程度上可以辅助部分细菌耐药特征的判断，其可靠性主要依赖于数据库中耐药基因与表型的匹配度、mNGS检出病原的基因覆盖度以及耐药基因细菌溯源的准确性[26]–[27]。不同细菌耐药基因与表型的一致性差异较大，部分耐药基因可存在携带而不表达的情况，如铜绿假单胞菌、阴沟肠杆菌染色体携带AmpC基因但其未必表达，AmpC基因检出不等同于耐药；而部分基因一旦检出则应高度考虑耐药的存在，如常见的碳青霉烯类耐药基因。常见耐药基因与表型一致性较高，且属于重点关注的耐药菌及其耐药基因包括：肠杆菌目细菌NDM、KPC基因-碳青霉烯类耐药，blaCTX-M基因-广谱头孢菌素耐药；金黄色葡萄球菌mecA基因-常见β内酰胺类耐药；鲍曼不动杆菌blaOXA-23基因-碳青霉烯耐药；屎肠球菌vanA或vanB基因-万古霉素耐药。

对于非无菌部位样本，如痰液、BALF等，多种微生物检出常见，其耐药基因的检出应考虑与病原的对应情况，例如mecA基因的检出可能出自金黄色葡萄球菌，但也可能来自凝固酶阴性葡萄球菌，需结合病原匹配情况进行解读。同时，值得注意的是，无耐药基因型检出不等于敏感，这往往与检出病原的序列低导致基因覆盖度不够未能检出耐药基因有关。

共识15：病原mNGS检测属于定性检测范畴，序列高低不等同于体内病原载量。

对于培养已转阴但感染仍控制不佳的患者，可尝试使用mNGS进行疗效评估，如病原仍可持续检出往往代表感染未能完全控制。但鉴于目前我国主要使用的病原mNGS检测手段仍属于定性检测水平，序列数高低不等同于病原载量，应审慎应用于疗效评价，同时应结合人源背景、测序数据量等综合评估。此外，对于不同样本之间因实验流程、人源比例均存在较大差异，不可直接进行序列数比较来评估疗效。

共识16：不推荐将mNGS阴性作为排除感染的标准。

尽管mNGS病原检出率高于传统微生物学检测，但仍存在漏检可能，常见原因主要包括：①标本中人源背景高，微生物核酸比例过低导致未能检出，例如脑脊液样本中病毒检出的灵敏度对标定量PCR检测存在漏检可能；②感染灶隐匿，无游离病原核酸释放入血，例如肝脾念珠菌病时，病原相对局限；③患者粒缺状态，病原主要以完整细胞形式存在，血液中无病原游离核酸存在；④感染病原细胞壁较厚，破壁程度不足导致漏检，例如隐球菌和结核分枝杆菌；⑤标本代表性不足或质量不佳。

总之，对于血液病患者，病原mNGS通过对样本快速高通量测序，可以获得更全面、相对无偏倚的病原信息，对感染病原诊断起到积极的作用，尤其对传统微生物学检测未覆盖到或检测周期较长、阳性率较低的病原可提高检出率。在临床应用中，考虑到其目前成本仍相对较高，应对检测适应证加以规范和限定，其主要应用领域仍然是急、危、重、难的患者感染诊治，避免过度使用。对于临床感染相关样本应首先完善传统微生物学检测，病理、无菌标本培养仍然是感染诊断的金标准，病原mNGS是对传统微生物学检测的有力补充和延展而非替代。病原mNGS的报告解读应充分评估检出微生物的致病性、流行病学、生物信息学信息，同时在全面结合患者临床特征的基础上进行综合判断。
